# 1*H*-Pyrazolo[4,3-*g*]benzothia­zol-7-amine

**DOI:** 10.1107/S1600536809012550

**Published:** 2009-04-18

**Authors:** José R. Camacho, Teresa González

**Affiliations:** a Laboratorio 233, Departamento de Química, Universidad Simon Bolivar (USB), Apartado 47206, Caracas 1080-A, Venezuela; b Centro de Química, Instituto Venezolano de Investigaciones Científicas (IVIC), Apartado 21827, Caracas 1020-A, Venezuela

## Abstract

The mol­ecule of the title compound, C_8_H_6_N_4_S, is almost planar [maximum deviation from the mean plane = 0.020 (1) Å for the S atom]. In the crystal, a supra­molecular three-dimensional arrangement arises from N—H⋯N hydrogen bonds and weak aromatic stacking interactions along the *a* axis [centroid–centroid separation = 3.582 (2) Å].

## Related literature

For background on DNA inter­calation agents, see: Cagnoli *et al.* (1968 ▶); Martínez & Chacón-García (2005 ▶); Chakrabarty *et al.* (2008 ▶). For further synthetic details, see: Salazar & Dorta (2004 ▶).
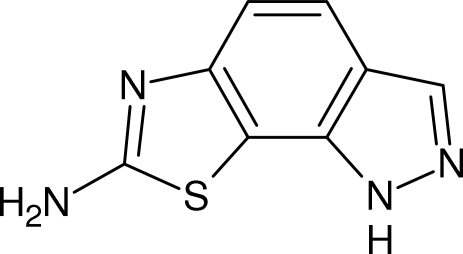


## Experimental

### 

#### Crystal data

C_8_H_6_N_4_S
*M* *_r_* = 190.23
Monoclinic, 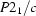

*a* = 4.499 (2) Å
*b* = 14.979 (8) Å
*c* = 12.112 (7) Å
β = 92.442 (19)°
*V* = 815.6 (7) Å^3^
*Z* = 4
Mo *K*α radiation
μ = 0.35 mm^−1^
*T* = 293 K
0.50 × 0.48 × 0.28 mm

#### Data collection

Rigaku AFC7S Mercury diffractometer
Absorption correction: multi-scan (Jacobson, 1998 ▶) *T*_min_ = 0.837, *T*_max_ = 0.904
8415 measured reflections
1564 independent reflections
1356 reflections with *I* > 2σ(*I*)
*R*_int_ = 0.027

#### Refinement

*R*[*F* ^2^ > 2σ(*F* ^2^)] = 0.043
*wR*(*F* ^2^) = 0.117
*S* = 1.12
1564 reflections
118 parameters
H-atom parameters constrained
Δρ_max_ = 0.21 e Å^−3^
Δρ_min_ = −0.39 e Å^−3^

### 

Data collection: *CrystalClear* (Rigaku, 2002 ▶); cell refinement: *CrystalClear*; data reduction: *CrystalStructure* (Rigaku/MSC, 2004 ▶); program(s) used to solve structure: *SHELXTL-NT* (Sheldrick, 2008 ▶); program(s) used to refine structure: *SHELXTL-NT*; molecular graphics: *SHELXTL-NT* and *DIAMOND* (Brandenburg, 1999 ▶); software used to prepare material for publication: *SHELXTL-NT* and *PLATON* (Spek, 2009 ▶).

## Supplementary Material

Crystal structure: contains datablocks I. DOI: 10.1107/S1600536809012550/hb2931sup1.cif

Structure factors: contains datablocks I. DOI: 10.1107/S1600536809012550/hb2931Isup2.hkl

Additional supplementary materials:  crystallographic information; 3D view; checkCIF report

## Figures and Tables

**Table 1:** Hydrogen-bond geometry (Å, °)

*D*—H⋯*A*	*D*—H	H⋯*A*	*D*⋯*A*	*D*—H⋯*A*
N2—H2⋯N3^i^	0.95	1.92	2.864 (3)	170
N4—H5⋯N1^ii^	0.98	2.19	3.123 (3)	158
N4—H6⋯N1^iii^	0.95	2.09	3.019 (3)	164

Symmetry codes: (i) 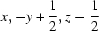; (ii) 
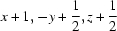
; (iii) 
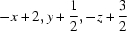
.

**Fig. 1.**
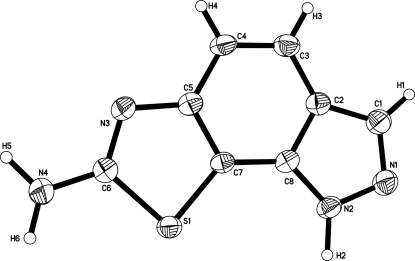
The molecular structure of (I), showing displacement elipsoids drawn at the 35% probability level and H atoms shown as spheres of arbitrary radius.

**Fig. 2.**
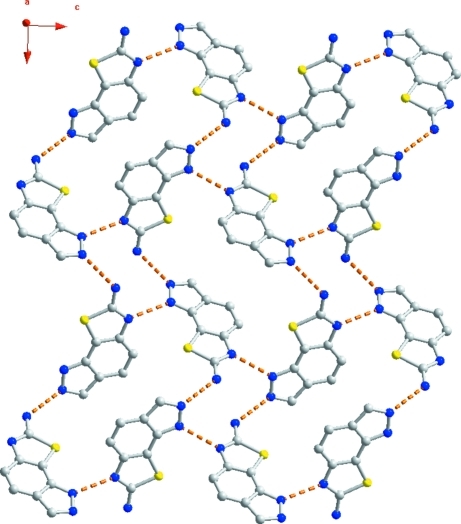
View of the hydrogen bonded layer in the bc plane in (I). The H atoms have been omitted for clarity and dashed lines indicate the donor···acceptor interactions for hydrogen bonds.

## supplementary crystallographic information

### Comment

Most of the significant advances against diseases have been made by designing and testing new structures, which are often heteroaromatic derivatives. Actually, the discovery of new compounds with antitumoral activity has become one of the most important goals in medicinal chemistry. One interesting group of potential chemotherapeutic agents includes molecules which interact with DNA like intercalator agents (Martínez & Chacón-García, 2005). Intercalation occurs when ligands of an appropriate size and chemical nature, fit themselves in between base pairs of DNA. Such ligands are mostly polycyclic, aromatic and planar. These kinds of agents are used in chemotherapeutic treatment to inhibit DNA replication in rapidly growing cancer cells. The title compound (I) is a polycyclic, aromatic and heterocyclic molecule and could be used to develop a new type of DNA intercalators agents (Chakrabarty *et al.* 2008).

The molecular structure is shown in figure 1, with its respective labels. The molecule adopts a conformation essenciality planar, with maximum deviation of the mean plane of 0.020 (1)Å for atom S1. The crystal structure of (I), consists of the self-assembly of the molecules through hydrogen bonding interactions of the kind N—H···N. The crystal packing (Fig. 2), consists of infinite chains in zigzag along the *b* axis generated by intermolecular interactions of hydrogen bond between the amino group and N atom of the imidazole ring [N1···N4 = 3.019 (3)Å]. These chains are connected through the interaction between the atoms N2 and N3 forming a two-dimensional wavy-like arrangement in the *bc* plane These layers are stacking through weak π–π interactions along the *a* axis (Cg3···Cg1, where Cg3 = C2/C3/C4/C5/C7/C8 and Cg1 = S1/C6/N3/C5/C7) together with an additional hydrogen bond lead to the formation of a three-dimensional hydrogen bonded network.

### Experimental

To a solution of 6-aminoindazole (2.00 g, 15 mmol) and NH_4_SCN (2.29 g, 30 mmol) in acetic acid (25 ml) was added dropwise pentylpyridinium tribromide (5.86 g, 15 mmol) (Salazar & Dorta, 2004). The resulting solution was stirred for 3 h at room temperature and then, 3 h at 80°C. The reaction mixture was then poured into ice (50 g), the precipitate was filtered and discarded. The resulting solution was neutralized with K_2_CO_3_, and the formed precipitate was filtered, washed with AcOEt and dried; to give a yellow powder corresponding to the title compound. Yield: 1.80 g (63%). The melting point (uncorrected) was measured with a Fischer-Johns micro hot-stage apparatus: d 563 K.

The yellow powder was dissolved in a minimum amount of 1,4-dioxane and the solution was left for several days at room temperature, during which the solution gradually reduced its volume to give light brown blocks of (I).

IR data [KBr pellets, (cm^-1^)]: 3367, 3314, 3181, 1629. ^1^H NMR [500 MHz, DMSO-d_6_, d (p.p.m.)]: 13.04 (brs, 1H, NH), 8.05 (s, 1H,), 7.57 (d, 1H, J = 6.84 Hz), 7.45 (brs, 2H, NH_2_), 7.21 (d, 1H, J = 6.88 Hz).13 C NMR [126 MHz, DMSO-d_6_, d (p.p.m.)]: 166.97 (C6), 152.21 (C5), 135.41 (C1), 134.99 (C2), 119.13 (C8), 118.01 (C3), 113.95 (C4), 109.39 (C7).

### Refinement

The N-bound H atoms were located in difference maps and refined as riding in their as found relative positions with *U*_iso_(H) = 1.2*U*_eq_(N). The C-bound H atoms were placed in idealized positions (C—H = 0.93–0.98 Å) and refined as riding with *U*_iso_(H) = 1.2*U*_eq_(C).

### Crystal data

**Table:**

C_8_H_6_N_4_S	*F*(000) = 392
*M**_r_* = 190.23	*D*_x_ = 1.549 Mg m^−^^3^
Monoclinic, *P*2_1_/*c*	Mo *K*α radiation, λ = 0.71070 Å
Hall symbol: -P 2ybc	Cell parameters from 5546 reflections
*a* = 4.499 (2) Å	θ = 1.4–27.8°
*b* = 14.979 (8) Å	µ = 0.35 mm^−^^1^
*c* = 12.112 (7) Å	*T* = 293 K
β = 92.442 (19)°	Block, light brown
*V* = 815.6 (7) Å^3^	0.50 × 0.48 × 0.28 mm
*Z* = 4	

### Data collection

**Table:**

Rigaku AFC7S Mercury diffractometer	1564 independent reflections
Radiation source: Normal-focus sealed tube	1356 reflections with *I* > 2σ(*I*)
graphite	*R*_int_ = 0.027
ω scans	θ_max_ = 27.8°, θ_min_ = 2.2°
Absorption correction: multi-scan (Jacobson, 1998)	*h* = −5→5
*T*_min_ = 0.837, *T*_max_ = 0.904	*k* = −17→17
8415 measured reflections	*l* = −10→13

### Refinement

**Table:**

Refinement on *F*^2^	Primary atom site location: structure-invariant direct methods
Least-squares matrix: full	Secondary atom site location: difference Fourier map
*R*[*F*^2^ > 2σ(*F*^2^)] = 0.043	Hydrogen site location: inferred from neighbouring sites
*wR*(*F*^2^) = 0.117	H-atom parameters constrained
*S* = 1.12	*w* = 1/[σ^2^(*F*_o_^2^) + (0.0537*P*)^2^ + 0.4259*P*] where *P* = (*F*_o_^2^ + 2*F*_c_^2^)/3
1564 reflections	(Δ/σ)_max_ = 0.001
118 parameters	Δρ_max_ = 0.21 e Å^−^^3^
0 restraints	Δρ_min_ = −0.38 e Å^−^^3^

### Special details

**Table:**

Geometry. All e.s.d.'s (except the e.s.d. in the dihedral angle between two l.s. planes) are estimated using the full covariance matrix. The cell e.s.d.'s are taken into account individually in the estimation of e.s.d.'s in distances, angles and torsion angles; correlations between e.s.d.'s in cell parameters are only used when they are defined by crystal symmetry. An approximate (isotropic) treatment of cell e.s.d.'s is used for estimating e.s.d.'s involving l.s. planes.
Refinement. Refinement of *F*^2^ against ALL reflections. The weighted *R*-factor *wR* and goodness of fit *S* are based on *F*^2^, conventional *R*-factors *R* are based on *F*, with *F* set to zero for negative *F*^2^. The threshold expression of *F*^2^ > σ(*F*^2^) is used only for calculating *R*-factors(gt) *etc*. and is not relevant to the choice of reflections for refinement. *R*-factors based on *F*^2^ are statistically about twice as large as those based on *F*, and *R*- factors based on ALL data will be even larger.

### Fractional atomic coordinates and isotropic or equivalent isotropic displacement parameters (Å^2^)

**Table:**

	*x*	*y*	*z*	*U*_iso_*/*U*_eq_	
S1	1.01959 (12)	0.29819 (4)	0.80928 (4)	0.0375 (2)	
N1	0.4558 (5)	0.03847 (13)	0.69844 (15)	0.0469 (5)	
N2	0.6362 (4)	0.11194 (12)	0.70868 (14)	0.0399 (5)	
H2	0.7275	0.1305	0.6432	0.048*	
N3	0.8801 (4)	0.31237 (12)	1.01512 (15)	0.0374 (4)	
N4	1.2153 (5)	0.42105 (13)	0.95801 (16)	0.0467 (5)	
H6	1.3202	0.4479	0.8999	0.056*	
H5	1.2408	0.4424	1.0345	0.056*	
C1	0.3414 (6)	0.02732 (16)	0.79596 (19)	0.0447 (6)	
H1	0.2103	−0.0183	0.8125	0.054*	
C2	0.4424 (5)	0.09335 (14)	0.87253 (17)	0.0364 (5)	
C3	0.3893 (5)	0.11384 (15)	0.98363 (18)	0.0425 (5)	
H3	0.2600	0.0790	1.0233	0.051*	
C4	0.5305 (5)	0.18567 (15)	1.03243 (18)	0.0408 (5)	
H4	0.4976	0.1996	1.1057	0.049*	
C5	0.7267 (5)	0.23892 (14)	0.97160 (16)	0.0352 (5)	
C6	1.0420 (5)	0.34903 (14)	0.94037 (17)	0.0354 (5)	
C7	0.7767 (5)	0.22046 (13)	0.86132 (16)	0.0319 (5)	
C8	0.6336 (5)	0.14676 (14)	0.81177 (16)	0.0333 (5)	

### Atomic displacement parameters (Å^2^)

**Table:**

	*U*^11^	*U*^22^	*U*^33^	*U*^12^	*U*^13^	*U*^23^
S1	0.0429 (4)	0.0374 (4)	0.0325 (4)	−0.0007 (2)	0.0051 (2)	0.00195 (19)
N1	0.0621 (14)	0.0372 (11)	0.0414 (11)	−0.0055 (9)	0.0022 (9)	−0.0022 (8)
N2	0.0526 (12)	0.0365 (10)	0.0308 (10)	−0.0003 (9)	0.0058 (8)	−0.0021 (7)
N3	0.0424 (11)	0.0372 (10)	0.0326 (10)	0.0027 (8)	0.0028 (8)	−0.0032 (7)
N4	0.0574 (13)	0.0421 (11)	0.0405 (11)	−0.0088 (9)	0.0014 (9)	0.0000 (8)
C1	0.0550 (15)	0.0365 (12)	0.0426 (13)	−0.0047 (10)	0.0028 (10)	0.0023 (9)
C2	0.0414 (13)	0.0312 (11)	0.0367 (11)	0.0028 (9)	0.0031 (9)	0.0043 (8)
C3	0.0489 (14)	0.0417 (13)	0.0375 (12)	−0.0020 (10)	0.0084 (10)	0.0051 (9)
C4	0.0503 (14)	0.0439 (13)	0.0290 (11)	0.0011 (10)	0.0091 (10)	0.0018 (8)
C5	0.0385 (12)	0.0340 (11)	0.0331 (11)	0.0060 (9)	0.0029 (8)	0.0005 (8)
C6	0.0363 (12)	0.0334 (11)	0.0364 (11)	0.0055 (9)	−0.0002 (8)	0.0019 (8)
C7	0.0346 (12)	0.0323 (11)	0.0289 (10)	0.0054 (9)	0.0038 (8)	0.0016 (8)
C8	0.0384 (12)	0.0312 (11)	0.0303 (10)	0.0083 (9)	0.0011 (8)	0.0007 (8)

### Geometric parameters (Å, °)

**Table:**

S1—C7	1.734 (2)	C1—C2	1.418 (3)
S1—C6	1.760 (2)	C1—H1	0.9300
N1—C1	1.319 (3)	C2—C8	1.405 (3)
N1—N2	1.370 (3)	C2—C3	1.411 (3)
N2—C8	1.354 (3)	C3—C4	1.371 (3)
N2—H2	0.9500	C3—H3	0.9300
N3—C6	1.307 (3)	C4—C5	1.419 (3)
N3—C5	1.391 (3)	C4—H4	0.9300
N4—C6	1.343 (3)	C5—C7	1.392 (3)
N4—H6	0.9529	C7—C8	1.400 (3)
N4—H5	0.9822		
			
C7—S1—C6	88.59 (10)	C2—C3—H3	120.4
C1—N1—N2	105.85 (18)	C3—C4—C5	120.3 (2)
C8—N2—N1	111.39 (18)	C3—C4—H4	119.9
C8—N2—H2	132.7	C5—C4—H4	119.9
N1—N2—H2	115.8	N3—C5—C7	115.03 (19)
C6—N3—C5	110.62 (18)	N3—C5—C4	123.89 (19)
C6—N4—H6	121.6	C7—C5—C4	121.1 (2)
C6—N4—H5	117.1	N3—C6—N4	124.3 (2)
H6—N4—H5	121.2	N3—C6—S1	115.53 (17)
N1—C1—C2	111.8 (2)	N4—C6—S1	120.12 (16)
N1—C1—H1	124.1	C5—C7—C8	118.54 (19)
C2—C1—H1	124.1	C5—C7—S1	110.22 (16)
C8—C2—C3	120.5 (2)	C8—C7—S1	131.23 (16)
C8—C2—C1	103.94 (19)	N2—C8—C7	132.6 (2)
C3—C2—C1	135.5 (2)	N2—C8—C2	107.05 (19)
C4—C3—C2	119.2 (2)	C7—C8—C2	120.32 (19)
C4—C3—H3	120.4		

### Hydrogen-bond geometry (Å, °)

**Table:**

*D*—H···*A*	*D*—H	H···*A*	*D*···*A*	*D*—H···*A*
N2—H2···N3^i^	0.95	1.92	2.864 (3)	170
N4—H5···N1^ii^	0.98	2.19	3.123 (3)	158
N4—H6···N1^iii^	0.95	2.09	3.019 (3)	164

Symmetry codes: (i) *x*, −*y*+1/2, *z*−1/2; (ii) *x*+1, −*y*+1/2, *z*+1/2; (iii) −*x*+2, *y*+1/2, −*z*+3/2.

## References

[bb1] Brandenburg, K. (1999). *DIAMOND* Crystal Impact GbR, Bonn,Germany.

[bb2] Cagnoli, N., Martani, A. & Fravolini, A. (1968). *Ann. Chim* pp. 823–837.

[bb3] Chakrabarty, M., Kundu, T., Arima, S. & Harigaya, Y. (2008). *Tetrahedron*, **64**, 6711–6723.

[bb4] Jacobson, R. (1998). Private communication to the Rigaku Corporation, Tokyo, Japan.

[bb5] Martínez, R. & Chacón-García, L. (2005). *Curr. Med. Chem* **12**, 127–151. 10.2174/0929867053363414 15638732

[bb6] Rigaku (2002). *CrystalClear* Rigaku Corporation, Tokyo, Japan.

[bb7] Rigaku/MSC (2004). *CrystalStructure* Rigaku/MSC, The Woodlands, Texas, USA.

[bb8] Salazar, J. & Dorta, R. (2004). *Synlett* **7**, 1318–1320.

[bb9] Sheldrick, G. M. (2008). *Acta Cryst.* A**64**, 112–122. 10.1107/S0108767307043930 18156677

[bb10] Spek, A. L. (2009). *Acta Cryst* D**65**, 148–155. 10.1107/S090744490804362X PMC2631630 19171970

